# When DNA barcoding and morphology mesh: Ceratopogonidae diversity in Finnmark, Norway

**DOI:** 10.3897/zookeys.463.7964

**Published:** 2014-12-12

**Authors:** Elisabeth Stur, Art Borkent

**Affiliations:** 1 Norwegian University of Science and Technology, NTNU University Museum, Department of Natural History, NO-7491 Trondheim, Norway; 2 Art Borkent, Research Associate of the Royal British Columbia Museum, the American Museum of Natural History, and Instituto Nacional de Biodiversidad, 691-8th Ave. SE, Salmon Arm, British Columbia, V1E 2C2, Canada

**Keywords:** *Atrichopogon*, *Forcipomyia*, *Dasyhelea*, *Culicoides*, *Brachypogon*, *Ceratopogon*, *Serromyia*, *Probezzia*, *Bezzia*, *Palpomyia*

## Abstract

DNA barcoding in Ceratopogonidae has been restricted to interpreting the medically and veterinary important members of *Culicoides* Latreille. Here the technique is utilised, together with morphological study, to interpret all members of the family in a select area. Limited sampling from the county of Finnmark in northernmost Norway indicated the presence of 54 species, including 14 likely new to science, 16 new to Norway, and one new to Europe. No species were previously recorded from this county. Only 93 species were known for all of Norway before this survey, indicating how poorly studied the group is. We evaluate and discuss morphological characters commonly used in identification of biting midges and relate species diagnoses to released DNA barcode data from 223 specimens forming 58 barcode clusters in our dataset. DNA barcodes and morphology were congruent for all species, except in three morphological species where highly divergent barcode clusters indicate the possible presence of cryptic species.

## Introduction

The Ceratopogonidae (biting midges) are generally small flies with a nearly worldwide distribution; the family includes 6,180 extant species in 111 genera (Borkent 2014a) but undoubtedly, many more undescribed species await discovery. Immatures are found in a wide array of aquatic, semiaquatic and moist terrestrial habitats. Female adults of many species in early lineages of the family suck blood from vertebrates or are ectoparasites on larger insects (e.g. wings of Odonata and Lepidoptera, caterpillars, phasmids). More derived lineages are predators of primarily nematocerous Diptera (e.g. Chironomidae) (Downes and Wirth 1981). Adults of both sexes imbibe nectar and/or honey dew and some are important pollinators of plants such as cocoa (Glendinning 1972). Numerous species of *Leptoconops* Skuse, *Forcipomyia* Meigen and *Culicoides* Latreille are pests of humans and livestock, having irritating bites and transmitting a wide array of viruses, protozoa and nematodes, including some important diseases (Borkent 2005).

Although Ceratopogonidae are common in almost all aquatic and semi-aquatic habitats, many species are small and members of some genera can be notoriously difficult to identify. The family is particularly poorly known taxonomically in Norway, in part due to very limited collecting and a general lack of experts over many years. Presently in Europe, the family has approximately half as many species as the Chironomidae, while in Norway this percentage is considerably lower (15%), as only 93 species of ceratopogonids have been recorded (Soot-Ryen 1943, Mehl 1996, Hagan et al. 2000, Thunes et al. 2004 and Szadziewski et al. 2012 ). Worldwide, however, there are about as many species of Ceratopogonidae (6,180) known as of Chironomidae (6235) (Borkent 2014a, Patrick Ashe pers. comm.). As this limited study of the ceratopogonid fauna of the far north shows, there are many more species of Ceratopogonidae actually present in Norway, with the strong expectation of further species both there and in more southerly habitats once these are systematically collected. Further to this, and particularly pertinent to studies of northern faunas, there have been only a few taxonomic studies comparing Old and New World Ceratopogonidae (Borkent and Bissett 1990; Borkent and Grogan 1995) and we therefore are uncertain about the true identity of some of these. There is an especially strong need to compare species of *Forcipomyia*, *Atrichopogon* Kieffer, *Dasyhelea* Kieffer, *Culicoides*, and *Brachypogon* Kieffer, all genera with numbers of species in the far north and which likely are more broadly distributed in the Holarctic than presently recognized.

Of all biting flies, the immatures of Ceratopogonidae are by far the most poorly known, with only limited regional keys to some larvae and pupae of some genera. To a distressing degree, the larvae of the subfamily Ceratopogoninae are morphologically similar and difficult to identify. The pupae are rich in characters and have been recently revised by Borkent (2014b).

DNA barcoding is defined as the use of short standardized sequences to identify specimens to species (Hebert et al. 2003). As a natural consequence, DNA barcodes can also be used to analyze species boundaries through genetic comparisons between similar taxa and provide an objective dataset to be used in the definition of species in addition to morphology, ecology and other species specific characteristics. The 5’ end of the mitochondrial gene cytochrome c oxidase subunit one (COI) is, since Hebert et al. (2003), regarded as the standard barcode region for animals and has been fairly widely used in Diptera (e.g. Ekrem et al. 2010; Renaud et al. 2012, Meiklejohn et al. 2012, Nagy et al. 2013). This marker is also used in the establishment of the Barcode Index Number (BIN) System, a DNA based registry for all animal species using operational taxonomic units as presumptive species (Ratnasingham and Hebert 2013). The use of COI-barcodes (or other molecular markers) to interpret species of Ceratopogonidae has barely begun and has focused on distinguishing those species of *Culicoides* implicated in the spread of diseases of domestic animals (e.g. Ander et al. 2013; Augot et al. 2013) as well as their hosts and parasites (Santiago-Alarcon et al. 2012). Our broader use here is the first to examine all the species of Ceratopogonidae at a given locality. Being a study of a high latitude fauna, the work provides ample opportunities to make future comparisons with the ceratopogonid fauna from elsewhere and especially from other localities in the northern Holarctic Region.

Neither Mehl (1996) in his overview of Norwegian *Culicoides* nor The Norwegian Biodiversity Information Centre (NBIC’s “Artsobservasjoner” and “Artskart”) have registered any Ceratopogonidae species from the county of Finnmark previous to our work.

## Material and methods

**Figure 1.**
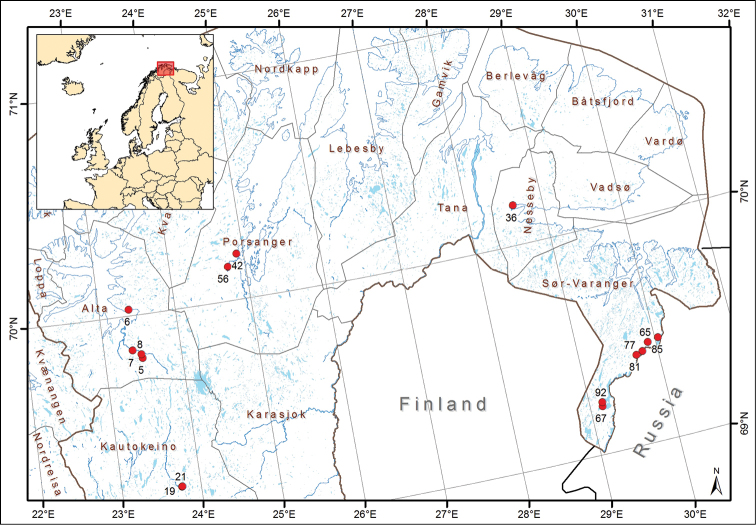
Sample sites for Ceratopogonidae in Finnmark, Norway, in 2010. Modified after Fig. 2 in Ekrem et al. (2012). Map by Marc Daverdin, NTNU University Museum.

Specimens were collected through a survey focusing on selected aquatic insect groups in Finnmark, the northernmost county of mainland Norway. More than 100 different sites were visited in three main trips during the season from June 11 to September 9, 2010 (Ekrem et al. 2012). Since the Ceratopogonidae were not a target group during the sampling, it is likely only a fraction of the existing species have been collected. The majority of Ceratopogonidae were retrieved from eight Malaise traps and only seven additional sites were sampled with sweep nets, dip nets or light trap (Fig. 1). All sample sites are described in Ekrem et al. (2012).

DNA barcodes were initially used to explore the unknown diversity of Ceratopogonidae from Finnmark. Several specimens of each morphotype were selected under a stereomicroscope and sampled for DNA analysis, typically by removing 1-3 legs. Tissues were shipped to the Biodiversity Institute of Ontario (BIO), Canada for sequencing of partial COI gene sequences. Mainly adult flies of both sexes were sequenced, but two larvae and one pupa were also included. COI amplification and sequencing followed standard protocols at the Canadian Centre for DNA Barcoding, BIO, including bi-directional Sanger sequencing. A list of barcoded material and all reference numbers are given in the Appendix; protocols, sequences, metadata and photographs of all specimens are available through the public project “Ceratopogonidae of Finnmark” [FICER] in the Barcode of Life Data Systems 3.0 (BOLD), (www.boldsystems.org, Ratnasingham and Hebert 2007).

Since slide mounting is generally needed for morphological species identification of biting midges, selected specimens (representing both sexes when available) from each cluster were slide mounted in Euparal©. The remaining un-mounted midges are preserved in 96% ethanol and stored in a -20 °C freezer. All specimens are deposited in the collection of the NTNU University Museum in Trondheim, Norway.

DNA barcodes from each genetic cluster (produced by the neighbor joining algorithm on Kimura 2-parameter genetic distances in BOLD) were compared with all COI sequences in BOLD and GenBank through the BOLD identification engine and GenBank’s MegaBLAST-algorithm (Morgulis et al. 2008) respectively. All instances which produced an identification different from our morphological identification are discussed in the taxonomic treatments below. We used MEGA 5.2 (Tamura et al. 2011) to generate the taxon ID-tree based on the neighbor joining algorithm from aligned COI sequences using partial deletion for areas with gaps and 1000 bootstrap replicates. The taxon ID-tree is not a phylogenetic hypothesis of the included taxa, but a graphic representation of barcode clusters based on genetic Kimura 2-parameter distances. Alignment was performed on protein sequences and was trivial as there were no observed indels and very high similarity on the amino acids level. Tools present in BOLD were used to produce a genetic distance summary and to perform a barcode gap analysis. All analyses were done using Kimura 2-parameter genetic distances (Kimura 1980).

Species were identified using taxonomic literature as referenced below (under each genus or species). Sources for Ceratopogonidae records in Norway were Soot-Ryen (1943), Mehl (1996), Hagan et al. (2000), Thunes et al. (2004), and Szadziewski et al. (2012). Comments on European distribution of Ceratopogonidae are based on data published in Fauna Europea (Szadziewski et al. 2012), for North America we relied on the summary distributions given by Borkent and Grogan (2009) and kept updated by the second author.

## Results

**Figure 2.**
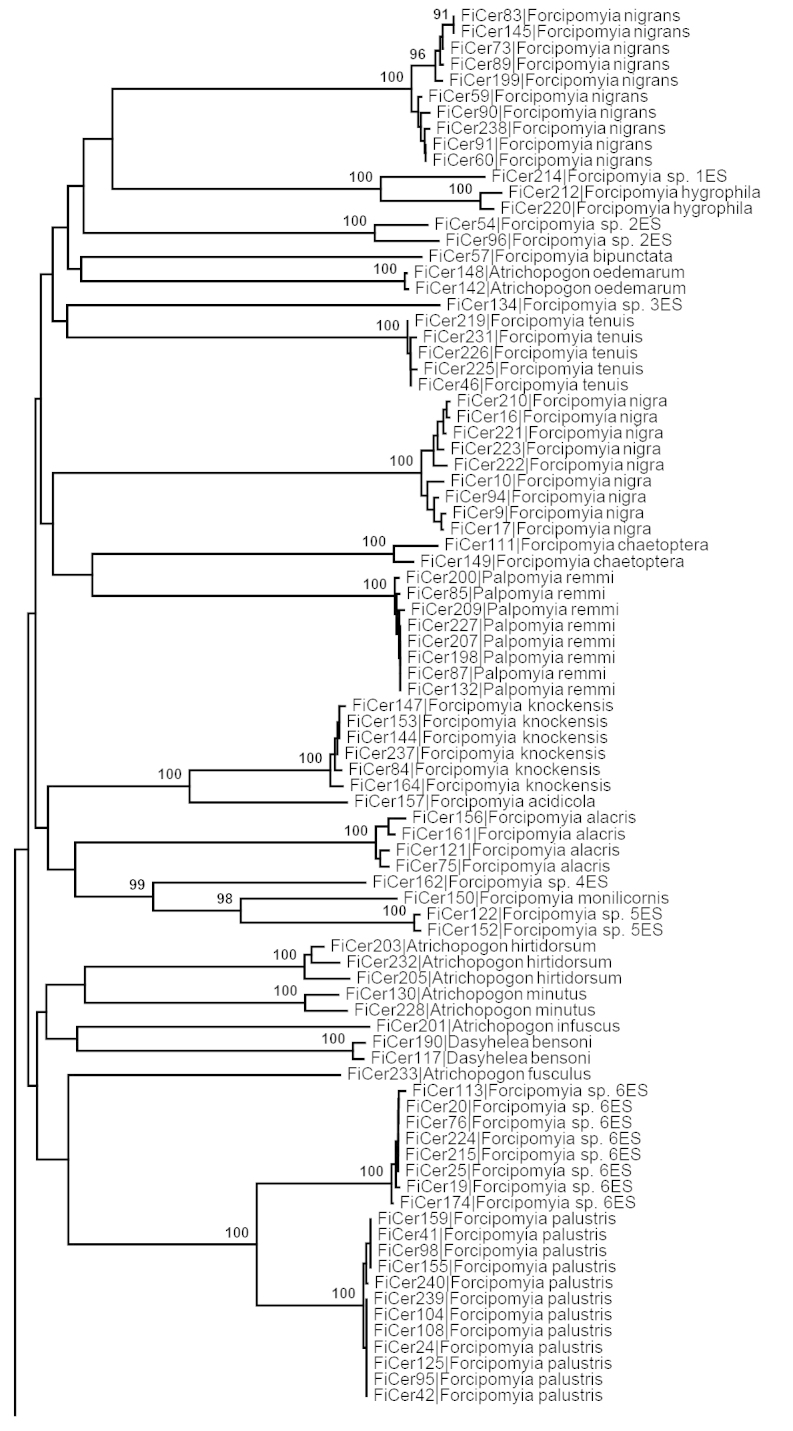

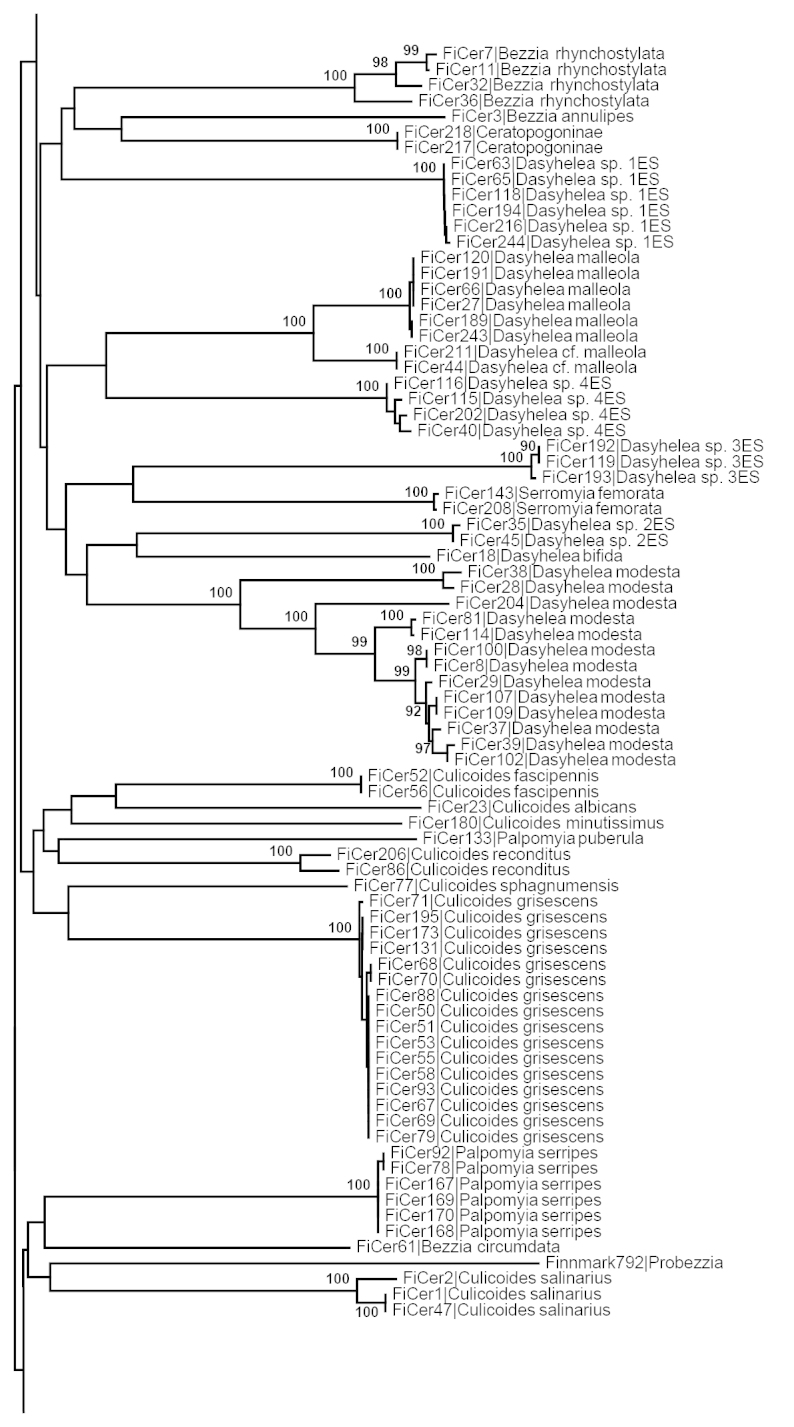

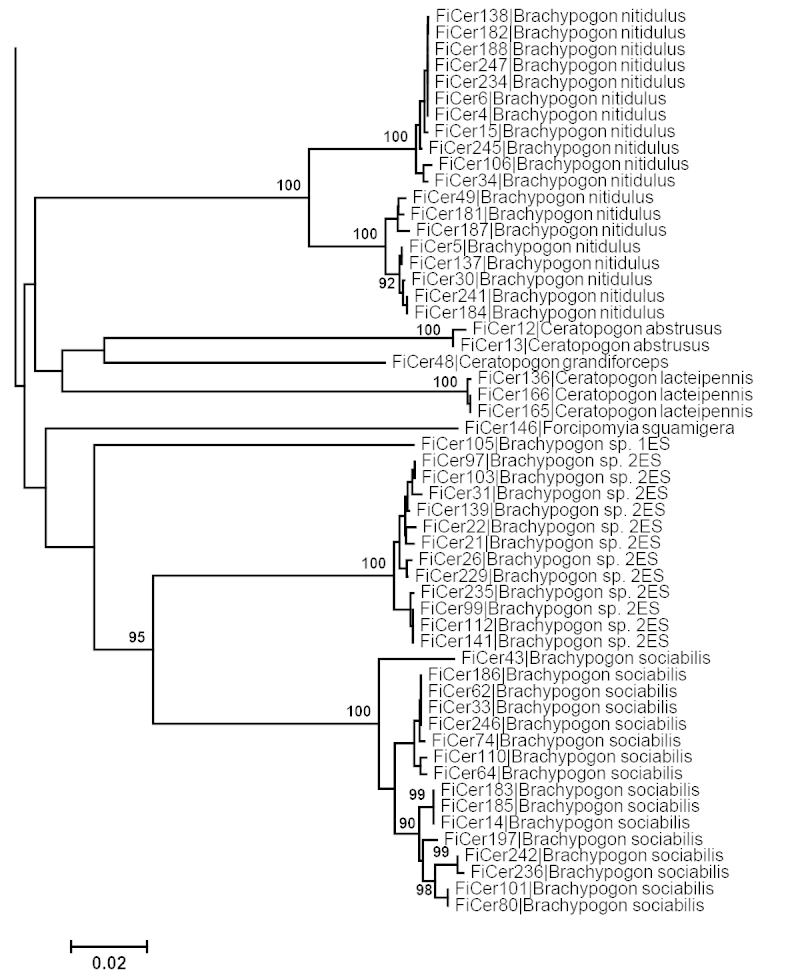
Taxon-ID tree of the studied Ceratopogonidae specimens based on the neighbor joining algorithm from aligned COI sequences using partial deletion for areas with gaps and 1000 bootstrap replicates in MEGA 5.2. All included sequences were longer than 500 bp. Bootstrap values shown on branches supported by more than 90% of the bootstrap replicates.

DNA barcodes were obtained from 223 specimens representing 54 morphological species (Table 1, Fig. 2). Thirty-eight species were represented by more than one specimen from Finnmark and showed a mean intraspecific Kimura 2-parameter distance of 1.6%. Maximum observed intraspecific distance for the complete dataset was considerably higher (11.9%) than the minimum observed interspecific divergence (5.8%). However, at least three morphological species contained multiple BINs (well separated barcode clusters) where cryptic species-level diversity may be present. Dasyhelea (Dicryptoscena) modesta (Winnertz, 1852) contains two BINs with mean intraspecific distance 4.9%, maximum intraspecific distance 11.9% and distance to nearest neighbor of a different morphospecies 16.9%. Dasyhelea (Dasyhelea) malleola Remm, 1962 contains four BINs with mean intraspecific distance 2.2%, maximum intraspecific distance 5.1% and distance to nearest neighbor of a different morphospecies 15.1%. Brachypogon (Isohelea) nitidulus (Edwards, 1921) contains two BINs with mean intraspecific distance 3.2%, maximum intraspecific distance 6.0% and distance to nearest neighbor of a different morphospecies 17.2%. Treating the multiple clusters of these three morphospecies as presumptive (cryptic) species, the maximum intraspecific distance for the whole dataset is 4.0% compared to 5.8% minimum interspecific distance, giving an overall barcode-gap of almost 2%.

**Table 1.** Distribution of Ceratopogonidae in Finnmark based on the revised Strand-system (Økland 1981). Species marked with an asterisk (*) are also known from North America (Borkent and Grogan 2009). Division of Finnmark in four regions according to the revised “Strand-system” (Økland 1981): FV = western Finnmark, FI = inner Finnmark, FN = northern Finnmark and FØ = eastern Finnmark.

	FV	FI	FN	FØ	previously recorded in Norway
Forcipomyiinae					
Atrichopogon (Atrichopogon) hirtidorsum Remm, 1961	x			x	
Atrichopogon (Atrichopogon) infuscus Goetghebuer, 1929	x				
Atrichopogon (Atrichopogon) minutus (Meigen, 1830) *	x				X
Atrichopogon (Lophomyidium) fusculus (Coquillett, 1901) *				x	
Atrichopogon (Meloehelea) oedemerarum Storå, 1939 *				x	
Forcipomyia (Euprojoannisia) alacris (Winnertz, 1852)				x	
Forcipomyia (Euprojoannisia) palustris (Meigen, 1804) *	x			x	X
Forcipomyia (Euprojoannisia) sp. 6ES nr. *palustris*				x	
Forcipomyia (Forcipomyia) bipunctata (Linneus, 1767) *				x	
Forcipomyia (Forcipomyia) squamigera Kieffer, 1916				x	X
Forcipomyia (Forcipomyia) sp. 2ES “*bipunctata* group”	x				
Forcipomyia (Forcipomyia) sp. 3ES “*bipunctata* group”	x				
Forcipomyia (Forcipomyia) hygrophila Kieffer, 1925 *				x	X
Forcipomyia (Forcipomyia) nigra (Winnertz, 1852)	x			x	X
Forcipomyia (Forcipomyia) nigrans Remm, 1962	x	x	x	x	X
Forcipomyia (Forcipomyia) tenuis (Winnertz, 1852)	x			x	
Forcipomyia (Forcipomyia) sp. 1ES				x	
Forcipomyia (Synthyridomyia) acidicola (Tokunaga, 1937) *				x	X
Forcipomyia (Synthyridomyia) knockensis Goetghebuer, 1938				x	
Forcipomyia (Thyridomyia) monilicornis (Coquillett, 1905) *				x	X
Forcipomyia (Thyridomyia) sp. 4ES				x	
Forcipomyia (Thyridomyia) sp. 5ES				x	
Forcipomyia (Trichohelea) chaetoptera Remm, 1962				x	
Dasyheleinae					
Dasyhelea (Dasyhelea) bensoni Edwards, 1933				x	X
Dasyhelea (Dasyhelea) malleola Remm, 1962 (2 cluster)				x	X
Dasyhelea (Dasyhelea) sp. 4ES nr. *bilineata*/*pallidiventris*	x			x	
Dasyhelea (Dicryptoscena) modesta (Winnertz, 1852)	x			x	X
Dasyhelea (Prokempia) sp. 1ES	x			x	
Dasyhelea (Pseudoculicoides) bifida Zilahi-Sebess, 1936 *				x	
Dasyhelea (Pseudoculicoides) sp. 2ES *mutabilis* group				x	
Dasyhelea (Pseudoculicoides) sp. 3ES *mutabilis* group				x	
Ceratopogoninae					
Culicoidini					
Culicoides (Beltramyia) sphagnumensis Williams, 1955 *				x	X
Culicoides (Beltramyia) salinarius Kieffer, 1914			x	x	
Culicoides (Culicoides) grisescens Edwards, 1939	x		x	x	X
Culicoides (Oecacta) albicans Winnertz, 1852				x	X
Culicoides (Silvaticulicoides) fascipennis (Stæger, 1839)				x	X
Culicoides (Wirthomyia) minutissimus (Zetterstedt, 1855)				x	
Culicoides (Wirthomyia) reconditus Campbell & Pelham-Clinton, 1960	x				X
Ceratopogonini					
Brachypogon (Isohelea) nitidulus (Edwards, 1921)			x	x	X
Brachypogon (Isohelea) sociabilis (Goetghebuer, 1920)				x	X
Brachypogon (Isohelea) sp.1ES				x	
Brachypogon (Isohelea) sp. 2ES nr. *norvegicus* (sp.n.?)				x	
*Ceratopogon abstrusus* Borkent & Grogan, 1995 *				x	
*Ceratopogon grandiforceps* (Kieffer, 1913)			x		
*Ceratopogon lacteipennis* Zetterstedt, 1838				x	X
*Serromyia femorata* (Meigen, 1804)	x			x	X
Johannsenomyiini					
*Probezzia* sp. (pupa)				x	
Palpomyiini					
*Bezzia annulipes* (Meigen, 1830) *				x	
*Bezzia circumdata* (Staeger, 1839) *				x	X
*Bezzia rhynchostylata* Remm, 1974				x	X
*Palpomyia puberula* Remm, 1976	x				
*Palpomyia remmi* Havelka, 1974	x				X
*Palpomyia serripes* (Meigen, 1818)				x	X
Ceratopogonidae gen. sp. 1ES (larvae) (*Bezzia* or *Palpomyia*)	x			x	

There are two additional morphospecies where the Refined Single Linkage (RESL) analysis in BOLD (Ratnasingham and Hebert 2013) produces multiple BINs but where we suspect no more than one species: Brachypogon (Isohelea) sociabilis (Goetghebuer, 1920) has four BINs, a mean intraspecific distance of 1.71%, maximum intraspecific distance of 4.0% and distance to nearest neighbor 13.5%. *Bezzia rhynchostylata* Remm, 1974 has three BINs, a mean intraspecific distance of 2.4%, maximum intraspecific distance 3.8% and distance to nearest neighbor 17.2%. Both morphology and comparatively low intraspecific distance in these species suggest that the RESL algortithm overestimates presumptive species (as BINs) for these taxa.

We also compared our DNA barcodes with the partial COI gene sequences Ander et al. (2013) provided for 37 named *Culicoides* species from Sweden. All *Culicoides* species we collected in Finnmark, except for *Culicoides minutissimus* (Zetterstedt, 1855), are represented in their study and our DNA barcodes match 98–100% with the sequences Ander et al. (2013) deposited in GenBank. Identification of *Culicoides salinarius* Kieffer, 1914 based on morphology is consistent to Ander et al.’s (2013) and not to Wenk et al.’s (2012) interpretation of the species. Voucher material for the COI-sequences published by Wenk et al. (2012) and Ander et al. (2013) was requested from the respective authors, but unfortunately not made available for examination. Thus, we were unable to confirm if the identifications correspond to our morphological interpretation of *Culicoides salinarius*.

Five of the sample sites collected 92% of the investigated specimens and all but one species were found at the five sites FinLoc65, FinLoc05, FinLoc08, FinLoc85, and FinLoc42 (Fig. 1, Ekrem et al. 2012). The most productive location in terms of Ceratopogonidae material was one locality in the eastern part of the county (FinLoc65, Malaise 7) in which 72% of all specimens treated were sampled and 41 of 55 species were found. The other Malaise traps collected from 1.4% to 8.4% of the specimens and 3–13 species, while the light trap at the research station (FinLoc85) collected 4.7% of the specimens and four species, including three species not collected elsewhere.

## Taxonomic discussion

The “ID” referred to below is the individual DNA barcode specimen ID and serves as a link between the DNA barcode in BOLD and the voucher specimen. The “FinLoc” number denotes the specific collecting sites shown in Figure 1.

### Forcipomyiinae

#### Atrichopogon

We collected adults of five species of *Atrichopogon* representing three subgenera.

##### Atrichopogon (Atrichopogon) hirtidorsum Remm, 1961

All three females of Atrichopogon (Atrichopogon) hirtidorsum key to *Atrichopogon fossicola* in Goetghebuer (1934) (*Atrichopogon fossicola* is listed as a synonym of *Atrichopogon fuscus*) and to *Atrichopogon hirtidorsum* in Remm (1961) based on the length of the scutal bristles.

**Material examined.** 3♀♀ (ID: FiCer203, FiCer205, FiCer232), 23 July–07 August 2010, FinLoc08, Malaise trap.

##### Atrichopogon (Atrichopogon) infuscus Goetghebuer, 1929

The single male of Atrichopogon (Atrichopogon) infuscus keys to *Atrichopogon infuscus* both in Goetghebuer (1934) and Remm (1961). The available descriptions of *Atrichopogon infuscus* and *Atrichopogon hirtidorsum* are very basic and we have not examined types of these species. Thus, more detailed taxonomic revision of these species may change the identity of our examined specimens.

**Material examined.** 1♂ (ID: FiCer201) 23 July–07 August 2010, FinLoc08, Malaise trap.

##### Atrichopogon (Atrichopogon) minutus (Meigen, 1830)

Of the five *Atrichopogon* species we collected, only Atrichopogon (Atrichopogon) minutus has been previously recorded in Norway. The species is easily recognizable both as males and females using Remm’s (1961) and Havelka’s (1976) descriptions.

**Material examined.** 1♂ (ID: FiCer228) 10–23 July 2010, FinLoc08, Malaise trap, 1♀ (ID: FiCer130) 23 July–07 August 2010, FinLoc05, Malaise trap.

##### Atrichopogon (Lophomyidium) fusculus (Coquillett, 1901)

The female of Atrichopogon (Lophomyidium) fusculus fits the description in Szadsziewski et al. (1996) and is the first species record in the subgenus Atrichopogon (Lophomyidium) Cordero in Norway.

**Material examined.** 1♀ (ID: FiCer233) 30 July–10 August 2010, FinLoc65, Malaise trap.

##### Atrichopogon (Meloehelea) oedemerarum Storå, 1939

The two males of Atrichopogon (Meloehelea) oedemerarum closely match Szadsziewski et al.’s (1995) description of the species.

**Material examined.** 2♂♂ (ID: FiCer142, FiCer148) 24 June–20 July 2010, FinLoc65, Malaise trap.

#### Forcipomyia

Within the genus *Forcipomyia* we found 18 species distributed in five subgenera (Table 1). For identifying the subgenera we used the key and definitions in Wirth and Ratanaworabhan (1978), Debenham (1987), and a key to the subgenera restricted to Fennoscandia and northern Europe (Borkent unpublished). Alwin and Szadziewksi (2013) recently published a key to the subgenera present in Poland and confirms subgeneric identifications here. Identification of *Forcipomyia* at the species level are mostly based on the key and figures in Remm (1962), however, additional literature is used in individual cases (see below).

##### Forcipomyia (Euprojoannisia) alacris (Winnertz, 1852)

Forcipomyia (Euprojoannisia) alacris has been previously recorded in Norway.

**Material examined.** 3♂♂ (ID: FiCer121, FiCer156, FiCer161) 24 June–20 July 2010; 1♂ (ID: FiCer75) 30 July–10 August 2010, all FinLoc65, Malaise trap.

##### Forcipomyia (Euprojoannisia) palustris (Meigen, 1804)

*Forcipomyia palustris* has been previously recorded in Norway. The males match the description of Szadziewski (1986).

**Material examined.** 3♂♂ (ID: FiCer24, FiCer104, FiCer108), and 3♀♀ (ID: FiCer41, FiCer42, FiCer98), 19–24 June 2010, 4♀♀ (ID: FiCer95, FiCer125, FiCer155, FiCer159) 24 June–20 July 2010, 2♀♀ (ID: FiCer239, FiCer240) 30 July–10 August 2010, all FinLoc65, Malaise trap.

##### Forcipomyia (Euprojoannisia) sp. 6ES nr. *palustris*

*Forcipomyia* sp. 6ES nr. *palustris* is a species morphologically similar to *Forcipomyia palustris* but differs in subtle differences in the male genitalia: The gonocoxal apodemes are narrower apically and with very short lateral projections and posteriorly the ventral prong is more slender and elongate.

**Material examined.** 4♂♂ (ID: FiCer19, FiCer20, FiCer25, FiCer113) 19–24 June 2010, 1♀ (ID: FiCer76) 30 July–10 August 2010, 1♀ (ID: FiCer174) 24 June–20 July 2010, all FinLoc65, Malaise trap, 1♀ (ID: FiCer215) 11–26 June 2010, FinLoc05, Malaise trap, 1♂ (ID: FiCer224) 19 June 2010, netting.

##### Forcipomyia (Forcipomyia) bipunctata (Linnaeus, 1767)

The single male was identified as *Forcipomyia bipunctata* following the description of this species by Szadziewski et al. (2007). Additionally, the key and figures in Remm (1962) were consulted. Szadziewski et al. (2007) revised the European *bipunctata* species group of the subgenus Forcipomyia (Forcipomyia) and included *Forcipomyia bipunctata*, *Forcipomyia squamigera*, *Forcipomyia ciliata* (Winnertz, 1852), and *Forcipomyia pulchrithorax* Edwards, 1924.

**Material examined.** 1♂ (ID: FiCer57) 08 September 2010, FinLoc85, light trap.

##### Forcipomyia (Forcipomyia) squamigera Kieffer, 1916

The single male was identified as Forcipomyia (Forcipomyia) squamigera based on the description of the species in Szadziewski et al. (2007).

**Material examined.** 1♂ (ID: FiCer146) 24 June–20 July 2010, FinLoc65, Malaise trap.

##### Forcipomyia (Forcipomyia) sp. 2ES *bipunctata* group

The females of *Forcipomyia* sp. 2ES have lanceolate setae on all tibiae and elongated seminal capsules. They seem to belong within the *bipunctata* group (Szadziewski et al. 2007). For species determination an association with male specimens is necessary. Whether these two specimens belong to one or two species is not clear. More material and associations are necessary for accurate determination.

**Material examined.** 2♀♀ (ID: FiCer54, FiCer96) 07 and 08 September 2010, FinLoc85, light trap.

##### Forcipomyia (Forcipomyia) sp. 3ES *bipunctata* group

The single female, *Forcipomyia* sp. 3ES, with lanceaolate setae on mid and hind tibia, fits within the *bipunctata* group (Szadziewski et al. 2007). The larger setae on fore tibia are missing (broken) and could be lanceolate or not. This specimen has a wing length of 1.7 mm, like the largest species of this group, Forcipomyia (Forcipomyia) ciliata (Winnertz, 1852).

**Material examined.** 1♀ (ID: FiCer134) 23 July–07 August 2010, FinLoc05, Malaise trap.

##### Forcipomyia (Forcipomyia) hygrophila Kieffer, 1925

*Forcipomyia hygrophila* has been previously recorded in Norway.

**Material examined.** 1♂ (ID: FiCer220) 19 June 2010, FinLoc77, netting, 1♀ (ID: FiCer212) 11–26 June 2010, FinLoc05, Malaise trap.

##### Forcipomyia (Forcipomyia) sp. 1ES

**Figure 3.**
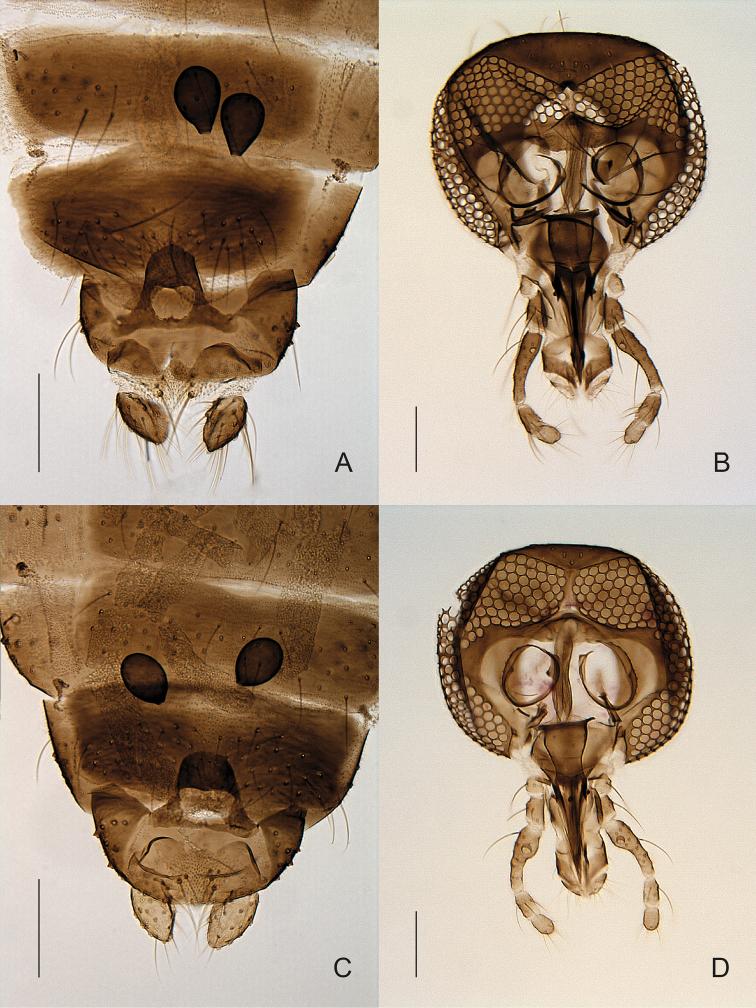
**A, B** *Forcipomyia hygrophila* female **A** terminalia, in ventral view **B** head, in anterior view **C, D** *Forcipomyia* sp. 1 female **C** terminalia, in ventral view **D** head, in anterior view.

The single female specimen of *Forcipomyia* sp. 1ES, is genetically relatively close to *Forcipomyia hygrophila* but easy to distinguish morphologically (e.g. by the shape of the palpus) (Fig. 3).

**Material examined.** 1♀ (ID: FiCer214) 11–26 June 2010, FinLoc05, Malaise trap.

##### Forcipomyia (Forcipomyia) nigra (Winnertz, 1852)

*Forcipomyia nigra* has been previously recorded in Norway.

**Material examined.** 4 ♂♂ (ID: FiCer9, FiCer10, FiCer16, FiCer17) 19–24 June 2010, 1♂ (ID: FiCer94) 24 June–20 July 2010, all FinLoc65, Malaise trap. 1♀ (ID: FiCer210) 11–26 June 2010, FinLoc05, Malaise trap, 2♂♂ (ID: FiCer221, FiCer222) 19 June 2010, FinLoc67, netting, 1♂ (ID: FiCer223) 19 June 2010, FinLoc81, netting.

##### Forcipomyia (Forcipomyia) nigrans Remm, 1962

*Forcipomyia nigrans* has been previously recorded in Norway.

**Material examined.** 1♂ (ID: FiCer59), 1♀ (ID: FiCer60) both 24 July–06 August 2010, FinLoc19, 1♂ (ID: FiCer90) and 1♀ (ID: FiCer91) both 24 July–06 August 2010, FinLoc21, 1♀ (ID: FiCer145) 24 June–20 July 2010, 1♀ (ID: FiCer83) 20–30 July 2010, 2♀♀ (ID: FiCer73, FiCer238) 30 July–10 August 2010, all four specimens FinLoc65, 1♀ (ID: FiCer89) 25 August–09 September 2010, FinLoc56, 1♀ (ID: FiCer199) 23 July–07 August 2010 FinLoc05, all Malaise trap.

##### Forcipomyia (Forcipomyia) tenuis (Winnertz, 1852)

*Forcipomyia tenuis* has not been recorded from Scandinavia before, but is known from many other European countries.

**Material examined.** 1♂ (ID: FiCer46) 17 June 2010, FinLoc36, 1♂ (ID: FiCer219) 19 June 2010, FinLoc77, 2♂♂ (ID: FiCer225, FiCer226) 13 June 2010, FinLoc06, all netting, 1♂ (ID: FiCer231) 20–30 July 2010, FinLoc65, Malaise trap.

##### Forcipomyia (Synthyridomyia) acidicola (Tokunaga, 1937)

Three species of the subgenus Forcipomyia (Synthyridomyia) are known from Europe. The single female specimen from Finnmark fits the diagnosis of the subgenus (Wirth and Ratanaworabhan 1978) and Tokunaga’s (1937) description of the species. *Forcipomyia acidicola* has been previously recorded in Norway (Thunes et al. 2004).

**Material examined.** 1♀ (ID: FiCer157) 24 June–20 July 2010, FinLoc65, Malaise trap.

##### Forcipomyia (Synthyridomyia) knockensis Goetghebuer, 1938

The identification of this species is based on the key of Remm (1962) and the redescription by Szadziewski (1983).

**Material examined.** 1♂ (ID: FiCer84) 20.–30. June 2010, 2♂♂ (ID: FiCer144, FiCer153) & 2♀♀ (ID: FiCer147, FiCer164) 24 June–20 July 2010, 1♀ (ID: FiCer237) 30 July–10 August 2010, all FinLoc65, Malaise trap.

##### Forcipomyia (Thyridomyia) monilicornis (Coquillett, 1905)

The species has been previously recorded in Norway.

**Material examined.** 1♂ (ID: FiCer150) 24 June–20 July 2010, FinLoc65, Malaise trap.

##### Forcipomyia (Thyridomyia) sp. 4ES

**Figure 4.**
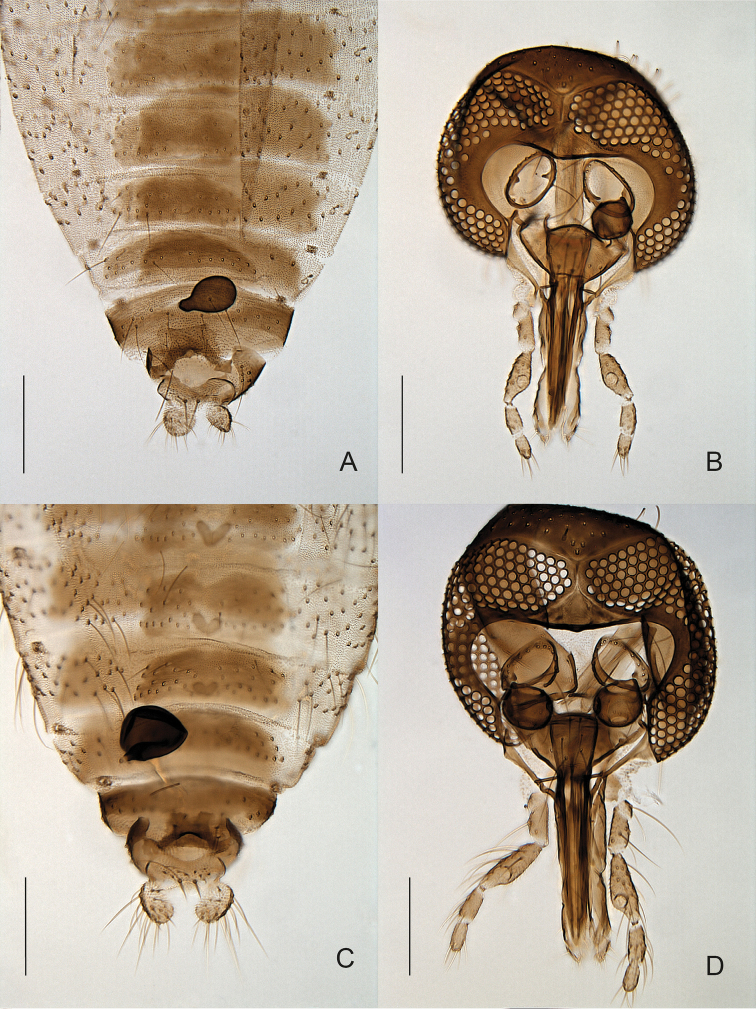
**A, B** *Forcipomyia* sp. 5 female **A** terminalia, in ventral view **B** head, in anterior view **C, D** *Forcipomyia* sp. 4 female **C** terminalia, in ventral view **D** head, in anterior view.

Three species within the subgenus Forcipomyia (Thyridomyia) could be distinguished: Forcipomyia (Thyridomyia) monilicornis, as well as two unnamed species Forcipomyia (Thyridomyia) sp. 4ES, and Forcipomyia (Thyridomyia) sp. 5ES. The two latter have only been collected as females (Fig. 4).

**Material examined.** 1♀ (ID: FiCer162) 24 June–20 July 2010, FinLoc65, Malaise trap.

##### Forcipomyia (Thyridomyia) sp. 5ES

**Material examined.** 2♀♀ (ID: FiCer122, FiCer152) 24 June–20 July 2010, FinLoc65, Malaise trap.

##### Forcipomyia (Trichohelea) chaetoptera Remm, 1962

The male and female key to Forcipomyia (Trichohelea) chaetoptera using Remm (1962).

**Material examined.** 1♂ (ID: FiCer111) 19–24 June 2010 & 1♀ (ID: FiCer149) 24 June–20 July 2010, both FinLoc65, Malaise trap.

### Dasyheleinae

#### Dasyhelea

The Polish species of this genus have recently been revised by Dominiak (2012) which included 30 of the 63 (Dominiak and Szadziewski 2010) known European species.

##### Dasyhelea (Dasyhelea) bensoni Edwards, 1933

*Dasyhelea bensoni* is not included in Dominiak’s (2012) key but the species is discussed within the description of *Dasyhelea pallidiventris* (Goetghebuer, 1931) in her work. An allocation of the two Finnmark females to the species is not definite since the palpal setae are missing on both specimens and no associated males have been collected. Dasyhelea (Dasyhelea) bensoni has been previously recorded in Norway.

**Material examined.** 2♀♀ (ID: FiCer117, FiCer190) 24 June–20 July 2010, FinLoc65, Malaise trap.

##### Dasyhelea (Dasyhelea) malleola Remm, 1962 (2 cluster)

**Figure 5.**
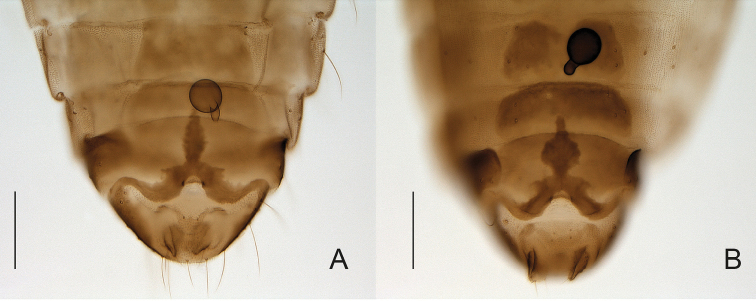
Female terminalia, in ventral view **A** Dasyhelea cf. malleola **B** *Dasyhelea malleola*.

There are two clusters of Dasyhelea (Dasyhelea) specimens, both including males and females, which key out to Dasyhelea (Dasyhelea) malleola and fit within the description for the species provided by Dominiak (2012). Whether or not these specimens are members of one or two species requires more material and a Holarctic revision of the genus. *Dasyhelea malleola* has been previously recorded in Norway. Between the males, no significant differences could be observed. The females however, differ in the shape of the posterior portion of sternite 9 (projecting anteriorly): subgenital fig elongate and vase shape in FiCer44 (Fig. 5) and widened in FiCer66, FiCer120, FiCer191 and FiCer243 (Fig. 5 and figure 39 in Dominiak (2012)). The spermatheca of specimen FiCer44 lacked pores and the extension was narrow; spermathecae of specimens FiCer191, FiCer120, FiCer243, and FiCer66 were with pores and the extension thicker). Since we only have one single male and female in the cluster of Dasyhelea cf. malleola it has to be confirmed with more material if the differences are consistent between the two forms.

**Material examined.** 1♂ (ID: FiCer27) and 1♀ (ID: FiCer44) 19–24 June 2010, 1♂ (ID: FiCer189) and 2♀♀ (ID: FiCer120, FiCer191) 24 June–20 July 2010, 1♀ (ID: FiCer243) 20–30 July 2010, 1♀ (ID: FiCer66), 30 July–08 August 2010, all FinLoc65, Malaise trap, 1♂ (ID: FiCer211) 11–26 June 2010, FinLoc05, Malaise trap.

##### Dasyhelea (Dasyhelea) sp. 4ES nr. *bilineata*/*pallidiventris*

The male and females of *Dasyhelea* sp. 4ES do not key to any of the species included in Dominiak’s (2012) key. Comparing the specimens with the provided species descriptions puts the species close to *Dasyhelea bilineata* Goetghebuer, 1920 and *Dasyhelea pallidiventris*.

**Material examined.** 1♀ (ID: FiCer40) 19–24 June 2010, 2♀♀ (ID: FiCer115, FiCer116) 24 June–20 July 2010, all FinLoc65, Malaise trap. 1♂ (ID: FiCer202) 23 July–07 August 2010, FiLoc08, Malaise trap.

##### Dasyhelea (Dicryptoscena) modesta (Winnertz, 1852)

Several specimens, both males and females, could be assigned to *Dasyhelea modesta* (Winnertz, 1852). They fit Dominiak’s (2012) interpretation of the species. The genetic distances for CO1 within the species cluster, however, can be as much as 10%, indicating the possibility of more than one species under this name.

**Material examined.** 8♂♂ (ID: FiCer8, FiCer28, FiCer29, FiCer100, FiCer102, FiCer107, FiCer109, FiCer114) and 3♀♀ (ID: FiCer37, FiCer38, FiCer39) 19–24 June 2010, 1♂ (ID: FiCer81) 20–30 July 2010 all FinLoc65, Malaise trap, 1♀ (ID: FiCer204) 23 July–07 August 2010, FiLoc08, Malaise trap.

##### Dasyhelea (Prokempia) sp. 1ES

All sampled *Dasyhelea* sp. 1ES specimens are females of the subgenus Dasyhelea (Prokempia). Species identification is only presently possible with males.

**Material examined.** 1♀ (ID: FiCer118) 19–24 June 2010, 1♀ (ID: FiCer194) 24 June–20 July 2010, 1♀ (ID: FiCer244) 20–30 July 2010, 2♀♀ (ID: FiCer63, FiCer65) 30 July–08 August 2010, all FinLoc65, Malaise trap, 1♀ (ID: FiCer216) 11–26 June 2010 FinLoc05, Malaise trap.

##### Dasyhelea (Pseudoculicoides) bifida Zilahi-Sebess, 1936

Of the three Dasyhelea (Pseudoculicoides) species collected, only *Dasyhelea bifida* could be named. Dasyhelea (Pseudoculicoides) sp. 2ES and Dasyhelea (Pseudoculicoides) sp. 3ES are members of the *mutabilis* group sensu Waugh and Wirth (1976). Associations to males are presently necessary to determine the nominal species.

**Material examined.** 1♂ (ID: FiCer18) 19–24 June 2010 FinLoc65, Malaise trap.

##### Dasyhelea (Pseudoculicoides) sp. 2ES

**Material examined.** 2♀♀ (ID: FiCer35, FiCer45) 19–24 June 2010 FinLoc65, Malaise trap.

##### Dasyhelea (Pseudoculicoides) sp. 3ES

**Material examined.** 3♀♀ (ID: FiCer119, FiCer) 24 June–20 July 2010 FinLoc65, Malaise trap.

### Ceratopogoninae

#### Culicoidini

##### Culicoides

Within the genus *Culicoides* we found seven species representing five subgenera. For identification, the keys and descriptions of Glukhova (2005) were used and, in addition, the key and descriptions in Campbell and Pelham-Clinton (1959) and Delécolle (1985) were consulted.

###### Culicoides (Beltramyia) sphagnumensis Williams, 1955

**Material examined.** 1♀ (FiCer77) 30 July–10 August 2010, FinLoc65, Malaise trap.

###### Culicoides (Beltramyia) salinarius Kieffer, 1914

The males of this species key to *Culicoides salinarius* in Delécolle (1985). The wings of these specimens have a single pale spot over r-m and CuA_2_ is dark, also features of females of this species.

**Material examined.** 2♂♂ (FiCer1, FiCer2) 19–24 June 2010, FinLoc65, Malaise trap.

1♂ (FiCer47) 15 June–02 July 2010, FinLoc42, Malaise trap.

###### Culicoides (Culicoides) grisescens Edwards, 1939

**Material examined.** 2♂♂ (FiCer93, FiCer173) 24 June–20 July 2010, 1♂ (FiCer79) 20–30 July 2010, 2♂♂ (FiCer68, FiCer70) and 3♀♀ (FiCer67, FiCer69, FiCer71) 30 July–08 August 2010, all FinLoc65, Malaise trap, 1♂ (FiCer195) and 1♀ (FiCer131) 23 July–07 August 2010, FinLoc05, Malaise trap, 1♂ (FiCer88) 25 August–09 September 2010, FinLoc56, Malaise trap, 3♂♂ (FiCer50, FiCer55, FiCer58) and 2♀♀ (FiCer51, FiCer53) 08 September 2010, FinLoc85, light trap.

###### Culicoides (Oecacta) albicans Winnertz, 1852

**Material examined.** 1♂ (FiCer23) 19–24 June 2010, FinLoc65, Malaise trap.

###### Culicoides (Silvaticulicoides) fascipennis (Stæger, 1839)

**Material examined.** 2♀♀ (FiCer52, FiCer56) 08 September 2010, FinLoc85, light trap.

###### Culicoides (Wirthomyia) minutissimus (Zetterstedt, 1855)

**Material examined.** 1♀ (FiCer180) 24 June–20 July 2010, FinLoc65, Malaise trap.

###### Culicoides (Wirthomyia) reconditus Campbell & Pelham-Clinton, 1960

**Material examined.** 1♂ (FiCer86) 10 - 23 July 2010, FinLoc05, Malaise trap, 1♀ (FiCer206) 23 July–07 August 2010, FinLoc08, Malaise trap.

#### Ceratopogonini

##### Genus *Brachypogon*

The *Brachypogon* species collected in Finnmark all belong to the subgenus Brachypogon (Isohelea).

###### Brachypogon (Isohelea) nitidulus (Edwards, 1921)

As mentioned above, there are two clearly divergent clusters of DNA barcodes from specimens identified as *Brachypogon nitidulus*, with a maximum Kimura 2-parameter distance of 5.98% (Figure 2). Specimens from the two clusters were collected at the same time and place and no morphological distinction is observed. We suspect that the fairly large observed COI divergence indicates possible cryptic species in this group. *Brachypogon nitidulus* has been previously recorded in Norway.

The male specimens of the cluster with FiCer04 have a relatively stout palpal segment 3, the males of cluster with FiCer05 have a more slender palpal segment 3.

**Material examined.** 7♂♂ (ID: FiCer4, FiCer5, FiCer6, FiCer15, FiCer30, FiCer106, FiCer245) and 2♀♀ (ID: FiCer34, FiCer247) 19–24 June 2010, 3♂♂ (ID: FiCer137, FiCer138, FiCer187) and 4♀♀ (ID: FiCer182, FiCer184, FiCer188) 24 June–20 July 2010, 1♀ (ID: FiCer241) 20–30 July 2010, 1♀ (ID: FiCer234) 30 July–10 August 2010, all FinLoc65, Malaise trap, 1♀ (ID: FiCer49) 15 June–02 July 2010, FinLoc42, Malaise trap.

###### Brachypogon (Isohelea) sociabilis (Goetghebuer, 1920)

*Brachypogon sociabilis* has been previously recorded in Norway.

**Material examined.** 3♂♂ (ID: FiCer14, FiCer101, FiCer110) and 3♀♀ (ID: FiCer33, FiCer43, FiCer246) 19–24 June 2010, 2♂♂ (ID: FiCer183, FiCer185) and 1♀ (ID: FiCer186) 24 June–20 July 2010, 1♂ (ID: FiCer80) and 1♀ (ID: FiCer242) 20–30 July 2010, 2♂♂ (ID: FiCer62, FiCer64) and 2♀♀ (ID: FiCer74, FiCer236) 30 July–10 August 2010, all FinLoc65, Malaise trap, 1♂ (ID: FiCer197) 23 July–07 August 2010, FinLoc05, Malaise trap.

###### Brachypogon (Isohelea) sp. 1ES

*Brachypogon* sp.1ES keys to Brachypogon (Isohelea) incompletus (Kieffer, 1925) in Szadziewski et al. (1994), but does not entirely fit their species description (e.g. differently shaped aedeagus).

**Material examined.** 1♂ (FiCer105) 19–24 June 2010, FinLoc65, Malaise trap.

###### Brachypogon (Isohelea) sp. 2ES

*Brachypogon* sp. **2ES** is a species similar to Brachypogon (Isohelea) norvegicus Szadziewski & Hagan, 2000 but with a differently shaped aedeagus. The species is possibly new to science.

**Material examined.** ♂♂ (FiCer21, FiCer22, FiCer26, FiCer31, FiCer99, FiCer112) 19–24 June 2010, ♂ (FiCer141) 24 June–20 July 2010, ♂ (FiCer229) 20–30 July 2010, 1♀ (FiCer235) 30 July–10 August 2010, all FinLoc65, Malaise trap.

##### Genus *Ceratopogon*

All identifications are based on the generic revision by Borkent and Grogan (1995).

###### *Ceratopogon abstrusus* Borkent & Grogan, 1995

*Ceratopogon abstrusus* was described by Borkent and Grogan (1995) from the Nearctic with a wide range from Alaska to northern Greenland and has been referred by them as “the most broadly distributed of all *Ceratopogon* species”. The record from Finnmark is the first for the Palearctic (other than northern Greenland).

**Material examined.** 2♂♂ (FiCer12, FiCer13) 19–24 June 2010, FinLoc65, Malaise trap.

###### *Ceratopogon grandiforceps* (Kieffer, 1913)

*Ceratopogon grandiforceps* from Finnmark is the first record for Fennoscandia.

**Material examined.** 1♂ (FiCer48) 15 June–02 July 2010, FinLoc42, Malaise trap.

###### *Ceratopogon lacteipennis* Zetterstedt, 1838

*Ceratopogon lacteipennis* has been previously recorded in northern Norway (Senja) and other localities in Norway (Thunes et al. 2004, Hagan et al. 2000).

**Material examined.** 3♂♂ (FiCer136, FiCer165, FiCer166) 24 June–20 July 2010, FinLoc65, Malaise trap.

##### Genus *Serromyia*

###### *Serromyia femorata* (Meigen, 1804)

This species has been previously recorded in Norway and was redescribed by Borkent and Bissett (1990).

**Material examined.** 1♂ (FiCer208) 10–23 July 2010, FinLoc08, Malaise trap, 1♀ (ID: FiCer143) 24 June–20 July 2010, FinLoc65, Malaise trap.

#### Johannsenomyiini

##### Genus *Probezzia*

###### *Probezzia* sp.

A single pupa was collected in a drift sample (see Fig. 9 in Ekrem et al. 2012).

The pupa from Finnmark is the first record of this genus for Norway. Three European species of *Probezzia* are Holarctic in distribution (Wirth 1971). The specimen was identified to genus using the key to genera by Borkent (2014b).

**Material examined.** 1 pupa (Finnmark792) 19 June 2010, FinLoc92, drift.

#### Palpomyiini

##### Genus *Bezzia*

###### *Bezzia annulipes* (Meigen, 1830)

The specimen from Finnmark fits the description in Remm (1974a) and Wirth et al. (1984) for this Holarctic species. This is the first record of the species in Norway.

**Material examined.** 1♂ (FiCer3) 19–24 June 2010, FinLoc65, Malaise trap.

###### *Bezzia circumdata* (Staeger, 1839)

The species keys to *Brachypogon solstitialis* (Winnertz, 1852) in Remm 1974a which is currently considered a synonym of *Bezzia circumdata*.

**Material examined.** 1♀ (FiCer61) 30 July–08 August 2010, FinLoc65, Malaise trap.

###### *Bezzia rhynchostylata* Remm, 1974

The species keys to *Bezzia rhynchostylata* in Remm 1974b.

**Material examined.** 2♂♂ (FiCer7, FiCer11) and 2♀♀ (FiCer32, FiCer36) 19–24 June 2010, FinLoc65, Malaise trap.

##### Genus *Palpomyia*

###### *Palpomyia puberula* Remm, 1976

The examined female keys to and fits the description of *Palpomyia puberula* in Remm (1976).

**Material examined.** 1♀ (FiCer133) 23 July–07 August 2010, FinLoc05, Malaise trap.

###### *Palpomyia remmi* Havelka, 1974

The Finnmark specimens fit the description by Havelka (1974). Krzywinski (1997) records the species for the first time in Norway and mentions that the species could be conspecific to the North American *Palpomyia canadensis* Grogan & Wirth, 1979. If the two species are conspecific, *Palpomyia remmi* would have a Holarctic distribution.

**Material examined.** 1♂ (FiCer227) 10–23 July 2010 and 3♂♂ (FiCer200, FiCer207, FiCer209) 23 July–07 August 2010, FinLoc08, Malaise trap, 2♀♀ (FiCer85, FiCer87) 10–23 July 2010 and 1♀ (FiCer132, FiCer198) 23 July–07 August 2010, FinLoc05, Malaise trap.

###### *Palpomyia serripes* (Meigen, 1818)

The examined males and females key to and fit the description of *Palpomyia serripes* in Remm (1976). The species seems to have a “north-south” rather than a circumpolar distribution.

**Material examined.** 1♂ (FiCer92) and 4♀♀ (FiCer167, FiCer168, Ficer169, FiCer170) 24 June–20 July 2010, 1♀ (FiCer78) 20–30 July 2010, all FinLoc65, Malaise trap.

### Ceratopogonidae gen. sp. 1ES

The larvae belong to either *Bezzia* or *Palpomyia*. For further identification association with the adult is required.

**Material examined.** 2 larvae (FiCer217, FiCer218) 14 June 2010, FinLoc07, benthos.

## Discussion

Our relatively cursory sampling of Ceratopogonidae revealed a startling 54 species within nine genera. Of these, 40 could be identified to previously named species, and 14 are apparently either undescribed or are close to previously known species. Considering that no Ceratopogonidae have been previously recorded from Finnmark, this is a substantial increase in numbers and reflects the poorly sampled and interpreted state of this diverse and common family in northern Norway. There are several impediments to our understanding this group in Finnmark. For example, much of our collecting, especially with hand nets, was not focused on Ceratopogonidae, which often require a less delicate sweeping mode than is best for Chironomidae. Most of our specimens were collected with Malaise traps, especially with the trap at locality FinLoc65. Even with these considerable limitations, we uncovered a substantial diversity. Certainly, with further concerted sampling in Finnmark, we would expect to find a significantly more diverse fauna than reported here.

A second impediment to understanding Ceratopogonidae in Finnmark, Norway and Europe in general is the major gaps in taxonomic revisions. For most genera, there are no inclusive European keys, based on examination of types and comparative material and most current revisions are regional or country specific. Even the continent-wide threat of Bluetongue and the Schmallenberg virus, resulting in millions of Euros in losses to livestock, has failed as an incentive to produce a comprehensive taxonomic analysis of the species of *Culicoides*, some of which act as vectors of these diseases. Further to this, very few revisions have compared Palaearctic and Nearctic species, especially important for northern taxa, and this has made an understanding of the distributions of many species uncertain. In some instances, it is very likely that some Palaearctic and Nearctic species, presently with different names, are actually conspecific.

To complete comprehensive revisions, authors should check all available types. This too is an impediment to our understanding of a number of genera. Many species names are floating because no one has examined the types since they were first described (in some cases over 150 years ago!).

Much of this reflects the general state of support for taxonomy, which is generally poor to non-existent. In the meantime, the Ceratopogonidae are a case in point for the value of future studies. Many species live in peripheral aquatic habitats (edges of streams, ponds and marshes) or in very small water bodies (springs, small pools), habitats that are often under extreme threat on our planet. A better understanding of the fauna of these habitats would reinforce the concept that they need to be protected.

One advantage of the present study, despite the lack of some species names, is that every investigated specimen is DNA barcoded and kept as a voucher in a public collection. This makes it possible to include them in further taxonomic studies, and to associate other life stages at a later point in time when obtained. For morphological species that are represented by more than one barcode cluster (such as *Dasyhelea modesta* or *Brachypogon nitidulus*), detailed reexamination of vouchers will be required to discover possible morphological traits that may distinguish new taxa. Moreover, as Anderson et al. (2013) found for the chironomid genus *Micropsectra*, detailed comparison of multiple life stages, ecology and nuclear molecular markers should clarify whether some of the highly divergent barcode clusters obtained in our study actually represent different biological species.

## Appendix

**Appendix:** Overview of sequenced Ceratopogonidae specimens and species from Finnmark.

Sample ID	Species	Life Stage	Locality	Collection Date	Collectors	GenBank Accession
FiCer203	Atrichopogon (Atrichopogon) hirtidorsum	adult	FinLoc08	07-Aug-2010	Ekrem et al.	KJ767865
FiCer205	Atrichopogon (Atrichopogon) hirtidorsum	adult	FinLoc08	07-Aug-2010	Ekrem et al.	KJ767866
FiCer232	Atrichopogon (Atrichopogon) hirtidorsum	adult	FinLoc08	07-Aug-2010	Ekrem et al.	KJ767867
FiCer201	Atrichopogon (Atrichopogon) infuscus	adult	FinLoc08	07-Aug-2010	Ekrem et al.	KJ767868
FiCer130	Atrichopogon (Atrichopogon) minutus	adult	FinLoc05	27-Aug-2010	Ekrem et al.	KJ767869
FiCer228	Atrichopogon (Atrichopogon) minutus	adult	FinLoc08	23-Jul-2010	Ekrem et al.	KJ767870
FiCer233	Atrichopogon (Lophomyidium) fusculus	adult	FinLoc65	10-Aug-2010	Ekrem et al.	KJ767864
FiCer148	Atrichopogon (Meleohelea) oedemarum	adult	FinLoc65	20-Jul-2010	Ekrem et al.	KJ767871
FiCer142	Atrichopogon (Meleohelea) oedemarum	adult	FinLoc65	20-Jul-2010	Ekrem et al.	KJ767872
FiCer156	Forcipomyia (Euprojoannisia) alacris	adult	FinLoc65	20-Jul-2010	Ekrem et al.	KJ768001
FiCer121	Forcipomyia (Euprojoannisia) alacris	adult	FinLoc65	20-Jul-2010	Ekrem et al.	KJ768002
FiCer75	Forcipomyia (Euprojoannisia) alacris	adult	FinLoc65	08-Jul-2010	Ekrem et al.	KJ768003
FiCer161	Forcipomyia (Euprojoannisia) alacris	adult	FinLoc65	20-Jul-2010	Ekrem et al.	KJ768004
FiCer239	Forcipomyia (Euprojoannisia) palustris	adult	FinLoc65	10-Aug-2010	Ekrem et al.	KJ768036
FiCer104	Forcipomyia (Euprojoannisia) palustris	adult	FinLoc65	24-Jun-2010	Ekrem et al.	KJ768037
FiCer108	Forcipomyia (Euprojoannisia) palustris	adult	FinLoc65	24-Jun-2010	Ekrem et al.	KJ768038
FiCer24	Forcipomyia (Euprojoannisia) palustris	adult	FinLoc65	24-Jun-2010	Ekrem et al.	KJ768039
FiCer155	Forcipomyia (Euprojoannisia) palustris	adult	FinLoc65	20-Jul-2010	Ekrem et al.	KJ768040
FiCer125	Forcipomyia (Euprojoannisia) palustris	adult	FinLoc65	20-Jul-2010	Ekrem et al.	KJ768041
FiCer95	Forcipomyia (Euprojoannisia) palustris	adult	FinLoc65	20-Jul-2010	Ekrem et al.	KJ768042
FiCer98	Forcipomyia (Euprojoannisia) palustris	adult	FinLoc65	24-Jun-2010	Ekrem et al.	KJ768043
FiCer240	Forcipomyia (Euprojoannisia) palustris	adult	FinLoc65	10-Aug-2010	Ekrem et al.	KJ768044
FiCer159	Forcipomyia (Euprojoannisia) palustris	adult	FinLoc65	20-Jul-2010	Ekrem et al.	KJ768045
FiCer41	Forcipomyia (Euprojoannisia) palustris	adult	FinLoc65	24-Jun-2010	Ekrem et al.	KJ768046
FiCer42	Forcipomyia (Euprojoannisia) palustris	adult	FinLoc65	24-Jun-2010	Ekrem et al.	KJ768047
FiCer25	Forcipomyia (Euprojoannisia) sp. 6ES	adult	FinLoc65	24-Jun-2010	Ekrem et al.	KJ768055
FiCer215	Forcipomyia (Euprojoannisia) sp. 6ES	adult	FinLoc05	26-Jun-2010	Ekrem et al.	KJ768056
FiCer174	Forcipomyia (Euprojoannisia) sp. 6ES	adult	FinLoc65	20-Jul-2010	Ekrem et al.	KJ768057
FiCer224	Forcipomyia (Euprojoannisia) sp. 6ES	adult	FinLoc81	19-Jun-2010	T. Ekrem, E. Stur	KJ768058
FiCer113	Forcipomyia (Euprojoannisia) sp. 6ES	adult	FinLoc65	24-Jun-2010	Ekrem et al.	KJ768059
FiCer76	Forcipomyia (Euprojoannisia) sp. 6ES	adult	FinLoc65	08-Jul-2010	Ekrem et al.	KJ768060
FiCer19	Forcipomyia (Euprojoannisia) sp. 6ES	adult	FinLoc65	24-Jun-2010	Ekrem et al.	KJ768061
FiCer20	Forcipomyia (Euprojoannisia) sp. 6ES	adult	FinLoc65	24-Jun-2010	Ekrem et al.	KJ768062
FiCer57	Forcipomyia (Forcipomyia) bipunctata	adult	FinLoc65	08-Sep-2010	T. Andersen	KJ768005
FiCer146	Forcipomyia (Forcipomyia) squamigera	adult	FinLoc65	20-Jul-2010	Ekrem et al.	KJ768063
FiCer54	Forcipomyia (Forcipomyia) sp. 2ES	adult	FinLoc85	08-Sep-2010	T. Andersen	KJ768049
FiCer96	Forcipomyia (Forcipomyia) sp. 2ES	adult	FinLoc85	07-Sep-2010	T. Andersen	KJ768050
FiCer134	Forcipomyia (Forcipomyia) sp. 3ES	adult	FinLoc05	27-Aug-2010	Ekrem et al.	KJ768051
FiCer212	Forcipomyia (Forcipomyia) hygrophila	adult	FinLoc05	26-Jun-2010	Ekrem et al.	KJ768008
FiCer220	Forcipomyia (Forcipomyia) hygrophila	adult	FinLoc77	19-Jun-2010	T. Ekrem, E. Stur	KJ768009
FiCer9	Forcipomyia (Forcipomyia) nigra	adult	FinLoc65	24-Jun-2010	Ekrem et al.	KJ768017
FiCer222	Forcipomyia (Forcipomyia) nigra	adult	FinLoc67	19-Jun-2010	T. Ekrem, E. Stur	KJ768018
FiCer221	Forcipomyia (Forcipomyia) nigra	adult	FinLoc67	19-Jun-2010	T. Ekrem, E. Stur	KJ768019
FiCer210	Forcipomyia (Forcipomyia) nigra	adult	FinLoc05	26-Jun-2010	Ekrem et al.	KJ768020
FiCer17	Forcipomyia (Forcipomyia) nigra	adult	FinLoc65	24-Jun-2010	Ekrem et al.	KJ768021
FiCer16	Forcipomyia (Forcipomyia) nigra	adult	FinLoc65	24-Jun-2010	Ekrem et al.	KJ768022
FiCer94	Forcipomyia (Forcipomyia) nigra	adult	FinLoc65	20-Jul-2010	Ekrem et al.	KJ768023
FiCer10	Forcipomyia (Forcipomyia) nigra	adult	FinLoc65	24-Jun-2010	Ekrem et al.	KJ768024
FiCer223	Forcipomyia (Forcipomyia) nigra	adult	FinLoc81	19-Jun-2010	T. Ekrem, E. Stur	KJ768025
FiCer83	Forcipomyia (Forcipomyia) nigrans	adult	FinLoc65	30-Jul-2010	Ekrem et al.	KJ768026
FiCer145	Forcipomyia (Forcipomyia) nigrans	adult	FinLoc65	20-Jul-2010	Ekrem et al.	KJ768027
FiCer91	Forcipomyia (Forcipomyia) nigrans	adult	FinLoc21	06-Aug-2010	Ekrem et al.	KJ768028
FiCer90	Forcipomyia (Forcipomyia) nigrans	adult	FinLoc21	06-Aug-2010	Ekrem et al.	KJ768029
FiCer89	Forcipomyia (Forcipomyia) nigrans	adult	FinLoc56	09-Sep-2010	Ekrem et al.	KJ768030
FiCer238	Forcipomyia (Forcipomyia) nigrans	adult	FinLoc65	10-Aug-2010	Ekrem et al.	KJ768031
FiCer199	Forcipomyia (Forcipomyia) nigrans	adult	FinLoc05	07-Aug-2010	Ekrem et al.	KJ768032
FiCer73	Forcipomyia (Forcipomyia) nigrans	adult	FinLoc65	08-Jul-2010	Ekrem et al.	KJ768033
FiCer60	Forcipomyia (Forcipomyia) nigrans	adult	FinLoc19	06-Aug-2010	Ekrem et al.	KJ768034
FiCer59	Forcipomyia (Forcipomyia) nigrans	adult	FinLoc19	06-Aug-2010	Ekrem et al.	KJ768035
FiCer231	Forcipomyia (Forcipomyia) tenuis	adult	FinLoc65	30-Jul-2010	Ekrem et al.	KJ768064
FiCer226	Forcipomyia (Forcipomyia) tenuis	adult	FinLoc06	13-Jun-2010	T. Ekrem, E. Stur	KJ768065
FiCer225	Forcipomyia (Forcipomyia) tenuis	adult	FinLoc06	13-Jun-2010	T. Ekrem, E. Stur	KJ768066
FiCer219	Forcipomyia (Forcipomyia) tenuis	adult	FinLoc77	19-Jun-2010	T. Ekrem, E. Stur	KJ768067
FiCer46	Forcipomyia (Forcipomyia) tenuis	adult	FinLoc36	17-Jun-2010	E. Stur	KJ768068
FiCer214	Forcipomyia (Forcipomyia) sp. 1ES	adult	FinLoc05	26-Jun-2010	Ekrem et al.	KJ768048
FiCer157	Forcipomyia (Synthyridomyia) acidicola	adult	FinLoc65	20-Jul-2010	Ekrem et al.	KJ768000
FiCer237	Forcipomyia (Synthyridomyia) knockensis	adult	FinLoc65	10-Aug-2010	Ekrem et al.	KJ768010
FiCer84	Forcipomyia (Synthyridomyia) knockensis	adult	FinLoc65	30-Jul-2010	Ekrem et al.	KJ768011
FiCer147	Forcipomyia (Synthyridomyia) knockensis	adult	FinLoc65	20-Jul-2010	Ekrem et al.	KJ768012
FiCer144	Forcipomyia (Synthyridomyia) knockensis	adult	FinLoc65	20-Jul-2010	Ekrem et al.	KJ768013
FiCer153	Forcipomyia (Synthyridomyia) knockensis	adult	FinLoc65	20-Jul-2010	Ekrem et al.	KJ768014
FiCer164	Forcipomyia (Synthyridomyia) knockensis	adult	FinLoc65	20-Jul-2010	Ekrem et al.	KJ768015
FiCer150	Forcipomyia (Thyridomyia) monilicornis	adult	FinLoc65	20-Jul-2010	Ekrem et al.	KJ768016
FiCer162	Forcipomyia (Thyridomyia) sp. 4ES	adult	FinLoc65	20-Jul-2010	Ekrem et al.	KJ768052
FiCer122	Forcipomyia (Thyridomyia) sp. 5ES	adult	FinLoc65	20-Jul-2010	Ekrem et al.	KJ768053
FiCer152	Forcipomyia (Thyridomyia) sp. 5ES	adult	FinLoc65	20-Jul-2010	Ekrem et al.	KJ768054
FiCer111	Forcipomyia (Trichohelea) chaetoptera	adult	FinLoc65	24-Jun-2010	Ekrem et al.	KJ768006
FiCer149	Forcipomyia (Trichohelea) chaetoptera	adult	FinLoc65	20-Jul-2010	Ekrem et al.	KJ768007
FiCer190	Dasyhelea (Dasyhelea) bensoni	adult	FinLoc65	20-Jul-2010	Ekrem et al.	KJ767961
FiCer117	Dasyhelea (Dasyhelea) bensoni	adult	FinLoc65	20-Jul-2010	Ekrem et al.	KJ767962
FiCer211	Dasyhelea (Dasyhelea) malleola	adult	FinLoc05	26-Jun-2010	Ekrem et al.	KJ767964
FiCer44	Dasyhelea (Dasyhelea) malleola	adult	FinLoc65	24-Jun-2010	Ekrem et al.	KJ767965
FiCer27	Dasyhelea (Dasyhelea) malleola	adult	FinLoc65	24-Jun-2010	Ekrem et al.	KJ767966
FiCer66	Dasyhelea (Dasyhelea) malleola	adult	FinLoc65	08-Jul-2010	Ekrem et al.	KJ767967
FiCer120	Dasyhelea (Dasyhelea) malleola	adult	FinLoc65	20-Jul-2010	Ekrem et al.	KJ767968
FiCer191	Dasyhelea (Dasyhelea) malleola	adult	FinLoc65	20-Jul-2010	Ekrem et al.	KJ767969
FiCer189	Dasyhelea (Dasyhelea) malleola	adult	FinLoc65	20-Jul-2010	Ekrem et al.	KJ767970
FiCer243	Dasyhelea (Dasyhelea) malleola	adult	FinLoc65	30-Jul-2010	Ekrem et al.	KJ767971
FiCer202	Dasyhelea (Dasyhelea) sp. 4ES	adult	FinLoc08	07-Aug-2010	Ekrem et al.	KJ767996
FiCer40	Dasyhelea (Dasyhelea) sp. 4ES	adult	FinLoc65	24-Jun-2010	Ekrem et al.	KJ767997
FiCer116	Dasyhelea (Dasyhelea) sp. 4ES	adult	FinLoc65	20-Jul-2010	Ekrem et al.	KJ767998
FiCer115	Dasyhelea (Dasyhelea) sp. 4ES	adult	FinLoc65	20-Jul-2010	Ekrem et al.	KJ767999
FiCer107	Dasyhelea (Dicryptoscena) modesta	adult	FinLoc65	24-Jun-2010	Ekrem et al.	KJ767972
FiCer81	Dasyhelea (Dicryptoscena) modesta	adult	FinLoc65	30-Jul-2010	Ekrem et al.	KJ767973
FiCer204	Dasyhelea (Dicryptoscena) modesta	adult	FinLoc08	07-Aug-2010	Ekrem et al.	KJ767974
FiCer39	Dasyhelea (Dicryptoscena) modesta	adult	FinLoc65	24-Jun-2010	Ekrem et al.	KJ767975
FiCer38	Dasyhelea (Dicryptoscena) modesta	adult	FinLoc65	24-Jun-2010	Ekrem et al.	KJ767976
FiCer37	Dasyhelea (Dicryptoscena) modesta	adult	FinLoc65	24-Jun-2010	Ekrem et al.	KJ767977
FiCer100	Dasyhelea (Dicryptoscena) modesta	adult	FinLoc65	24-Jun-2010	Ekrem et al.	KJ767978
FiCer29	Dasyhelea (Dicryptoscena) modesta	adult	FinLoc65	24-Jun-2010	Ekrem et al.	KJ767979
FiCer102	Dasyhelea (Dicryptoscena) modesta	adult	FinLoc65	24-Jun-2010	Ekrem et al.	KJ767980
FiCer28	Dasyhelea (Dicryptoscena) modesta	adult	FinLoc65	24-Jun-2010	Ekrem et al.	KJ767981
FiCer8	Dasyhelea (Dicryptoscena) modesta	adult	FinLoc65	24-Jun-2010	Ekrem et al.	KJ767982
FiCer114	Dasyhelea (Dicryptoscena) modesta	adult	FinLoc65	24-Jun-2010	Ekrem et al.	KJ767983
FiCer109	Dasyhelea (Dicryptoscena) modesta	adult	FinLoc65	24-Jun-2010	Ekrem et al.	KJ767984
FiCer63	Dasyhelea (Prokempia) sp. 1ES	adult	FinLoc65	08-Jul-2010	Ekrem et al.	KJ767985
FiCer65	Dasyhelea (Prokempia) sp. 1ES	adult	FinLoc65	08-Jul-2010	Ekrem et al.	KJ767986
FiCer118	Dasyhelea (Prokempia) sp. 1ES	adult	FinLoc65	20-Jul-2010	Ekrem et al.	KJ767987
FiCer194	Dasyhelea (Prokempia) sp. 1ES	adult	FinLoc65	20-Jul-2010	Ekrem et al.	KJ767988
FiCer216	Dasyhelea (Prokempia) sp. 1ES	adult	FinLoc05	26-Jun-2010	Ekrem et al.	KJ767989
FiCer244	Dasyhelea (Prokempia) sp. 1ES	adult	FinLoc65	30-Jul-2010	Ekrem et al.	KJ767990
FiCer18	Dasyhelea (Pseudoculicoides) bifida	adult	FinLoc65	24-Jun-2010	Ekrem et al.	KJ767963
FiCer35	Dasyhelea (Pseudoculicoides) sp. 2ES	adult	FinLoc65	24-Jun-2010	Ekrem et al.	KJ767991
FiCer45	Dasyhelea (Pseudoculicoides) sp. 2ES	adult	FinLoc65	24-Jun-2010	Ekrem et al.	KJ767992
FiCer193	Dasyhelea (Pseudoculicoides) sp. 3ES	adult	FinLoc65	20-Jul-2010	Ekrem et al.	KJ767993
FiCer192	Dasyhelea (Pseudoculicoides) sp. 3ES	adult	FinLoc65	20-Jul-2010	Ekrem et al.	KJ767994
FiCer119	Dasyhelea (Pseudoculicoides) sp. 3ES	adult	FinLoc65	20-Jul-2010	Ekrem et al.	KJ767995
FiCer1	Culicoides (Beltramyia) salinarius	adult	FinLoc65	24-Jun-2010	Ekrem et al.	KJ767957
FiCer2	Culicoides (Beltramyia) salinarius	adult	FinLoc65	24-Jun-2010	Ekrem et al.	KJ767958
FiCer47	Culicoides (Beltramyia) salinarius	adult	FinLoc42	02-Jul-2010	Ekrem et al.	KJ767959
FiCer77	Culicoides (Beltramyia) sphagnumensis	adult	FinLoc65	08-Jul-2010	Ekrem et al.	KJ767960
FiCer88	Culicoides (Culicoides) grisescens	adult	FinLoc56	09-Sep-2010	Ekrem et al.	KJ767938
FiCer131	Culicoides (Culicoides) grisescens	adult	FinLoc05	27-Aug-2010	Ekrem et al.	KJ767939
FiCer195	Culicoides (Culicoides) grisescens	adult	FinLoc05	07-Aug-2010	Ekrem et al.	KJ767940
FiCer50	Culicoides (Culicoides) grisescens	adult	FinLoc85	08-Sep-2010	T. Andersen	KJ767941
FiCer51	Culicoides (Culicoides) grisescens	adult	FinLoc85	08-Sep-2010	T. Andersen	KJ767942
FiCer53	Culicoides (Culicoides) grisescens	adult	FinLoc85	08-Sep-2010	T. Andersen	KJ767943
FiCer55	Culicoides (Culicoides) grisescens	adult	FinLoc85	08-Sep-2010	T. Andersen	KJ767944
FiCer58	Culicoides (Culicoides) grisescens	adult	FinLoc85	08-Sep-2010	T. Andersen	KJ767945
FiCer93	Culicoides (Culicoides) grisescens	adult	FinLoc65	20-Jul-2010	Ekrem et al.	KJ767946
FiCer67	Culicoides (Culicoides) grisescens	adult	FinLoc65	08-Jul-2010	Ekrem et al.	KJ767947
FiCer68	Culicoides (Culicoides) grisescens	adult	FinLoc65	08-Jul-2010	Ekrem et al.	KJ767948
FiCer69	Culicoides (Culicoides) grisescens	adult	FinLoc65	08-Jul-2010	Ekrem et al.	KJ767949
FiCer70	Culicoides (Culicoides) grisescens	adult	FinLoc65	08-Jul-2010	Ekrem et al.	KJ767950
FiCer71	Culicoides (Culicoides) grisescens	adult	FinLoc65	08-Jul-2010	Ekrem et al.	KJ767951
FiCer173	Culicoides (Culicoides) grisescens	adult	FinLoc65	20-Jul-2010	Ekrem et al.	KJ767952
FiCer79	Culicoides (Culicoides) grisescens	adult	FinLoc65	30-Jul-2010	Ekrem et al.	KJ767953
FiCer23	Culicoides (Oecacta) albicans	adult	FinLoc65	24-Jun-2010	Ekrem et al.	KJ767935
FiCer52	Culicoides (Silvaticulicoides) fascipennis	adult	FinLoc85	08-Sep-2010	T. Andersen	KJ767936
FiCer56	Culicoides (Silvaticulicoides) fascipennis	adult	FinLoc85	08-Sep-2010	T. Andersen	KJ767937
FiCer180	Culicoides (Wirthomyia) minutissimus	adult	FinLoc65	20-Jul-2010	Ekrem et al.	KJ767954
FiCer206	Culicoides (Wirthomyia) reconditus	adult	FinLoc08	07-Aug-2010	Ekrem et al.	KJ767955
FiCer86	Culicoides (Wirthomyia) reconditus	adult	FinLoc05	23-Jul-2010	Ekrem et al.	KJ767956
FiCer4	Brachypogon (Isohelea) nitidulus	adult	FinLoc65	24-Jun-2010	Ekrem et al.	KJ767879
FiCer5	Brachypogon (Isohelea) nitidulus	adult	FinLoc65	24-Jun-2010	Ekrem et al.	KJ767880
FiCer6	Brachypogon (Isohelea) nitidulus	adult	FinLoc65	24-Jun-2010	Ekrem et al.	KJ767881
FiCer106	Brachypogon (Isohelea) nitidulus	adult	FinLoc65	24-Jun-2010	Ekrem et al.	KJ767882
FiCer137	Brachypogon (Isohelea) nitidulus	adult	FinLoc65	20-Jul-2010	Ekrem et al.	KJ767883
FiCer241	Brachypogon (Isohelea) nitidulus	adult	FinLoc65	30-Jul-2010	Ekrem et al.	KJ767884
FiCer138	Brachypogon (Isohelea) nitidulus	adult	FinLoc65	20-Jul-2010	Ekrem et al.	KJ767885
FiCer30	Brachypogon (Isohelea) nitidulus	adult	FinLoc65	24-Jun-2010	Ekrem et al.	KJ767886
FiCer234	Brachypogon (Isohelea) nitidulus	adult	FinLoc65	10-Aug-2010	Ekrem et al.	KJ767887
FiCer15	Brachypogon (Isohelea) nitidulus	adult	FinLoc65	24-Jun-2010	Ekrem et al.	KJ767888
FiCer247	Brachypogon (Isohelea) nitidulus	adult	FinLoc65	24-Jun-2010	Ekrem et al.	KJ767889
FiCer188	Brachypogon (Isohelea) nitidulus	adult	FinLoc65	20-Jul-2010	Ekrem et al.	KJ767890
FiCer187	Brachypogon (Isohelea) nitidulus	adult	FinLoc65	20-Jul-2010	Ekrem et al.	KJ767891
FiCer49	Brachypogon (Isohelea) nitidulus	adult	FinLoc42	02-Jul-2010	Ekrem et al.	KJ767892
FiCer245	Brachypogon (Isohelea) nitidulus	adult	FinLoc65	24-Jun-2010	Ekrem et al.	KJ767893
FiCer184	Brachypogon (Isohelea) nitidulus	adult	FinLoc65	20-Jul-2010	Ekrem et al.	KJ767894
FiCer181	Brachypogon (Isohelea) nitidulus	adult	FinLoc65	20-Jul-2010	Ekrem et al.	KJ767895
FiCer182	Brachypogon (Isohelea) nitidulus	adult	FinLoc65	20-Jul-2010	Ekrem et al.	KJ767896
FiCer34	Brachypogon (Isohelea) nitidulus	adult	FinLoc65	24-Jun-2010	Ekrem et al.	KJ767897
FiCer110	Brachypogon (Isohelea) sociabilis	adult	FinLoc65	24-Jun-2010	Ekrem et al.	KJ767898
FiCer246	Brachypogon (Isohelea) sociabilis	adult	FinLoc65	24-Jun-2010	Ekrem et al.	KJ767899
FiCer242	Brachypogon (Isohelea) sociabilis	adult	FinLoc65	30-Jul-2010	Ekrem et al.	KJ767900
FiCer14	Brachypogon (Isohelea) sociabilis	adult	FinLoc65	24-Jun-2010	Ekrem et al.	KJ767901
FiCer236	Brachypogon (Isohelea) sociabilis	adult	FinLoc65	10-Aug-2010	Ekrem et al.	KJ767902
FiCer183	Brachypogon (Isohelea) sociabilis	adult	FinLoc65	20-Jul-2010	Ekrem et al.	KJ767903
FiCer101	Brachypogon (Isohelea) sociabilis	adult	FinLoc65	24-Jun-2010	Ekrem et al.	KJ767904
FiCer33	Brachypogon (Isohelea) sociabilis	adult	FinLoc65	24-Jun-2010	Ekrem et al.	KJ767905
FiCer185	Brachypogon (Isohelea) sociabilis	adult	FinLoc65	20-Jul-2010	Ekrem et al.	KJ767906
FiCer43	Brachypogon (Isohelea) sociabilis	adult	FinLoc65	24-Jun-2010	Ekrem et al.	KJ767907
FiCer186	Brachypogon (Isohelea) sociabilis	adult	FinLoc65	20-Jul-2010	Ekrem et al.	KJ767908
FiCer62	Brachypogon (Isohelea) sociabilis	adult	FinLoc65	08-Jul-2010	Ekrem et al.	KJ767909
FiCer64	Brachypogon (Isohelea) sociabilis	adult	FinLoc65	08-Jul-2010	Ekrem et al.	KJ767910
FiCer74	Brachypogon (Isohelea) sociabilis	adult	FinLoc65	08-Jul-2010	Ekrem et al.	KJ767911
FiCer80	Brachypogon (Isohelea) sociabilis	adult	FinLoc65	30-Jul-2010	Ekrem et al.	KJ767912
FiCer197	Brachypogon (Isohelea) sociabilis	adult	FinLoc05	07-Aug-2010	Ekrem et al.	KJ767913
FiCer105	Brachypogon (Isohelea) sp. 1ES	adult	FinLoc65	24-Jun-2010	Ekrem et al.	KJ767914
FiCer97	Brachypogon (Isohelea) sp. 2ES	adult	FinLoc65	24-Jun-2010	Ekrem et al.	KJ767915
FiCer99	Brachypogon (Isohelea) sp. 2ES	adult	FinLoc65	24-Jun-2010	Ekrem et al.	KJ767916
FiCer103	Brachypogon (Isohelea) sp. 2ES	adult	FinLoc65	24-Jun-2010	Ekrem et al.	KJ767917
FiCer26	Brachypogon (Isohelea) sp. 2ES	adult	FinLoc65	24-Jun-2010	Ekrem et al.	KJ767918
FiCer112	Brachypogon (Isohelea) sp. 2ES	adult	FinLoc65	24-Jun-2010	Ekrem et al.	KJ767919
FiCer22	Brachypogon (Isohelea) sp. 2ES	adult	FinLoc65	24-Jun-2010	Ekrem et al.	KJ767920
FiCer229	Brachypogon (Isohelea) sp. 2ES	adult	FinLoc65	30-Jul-2010	Ekrem et al.	KJ767921
FiCer21	Brachypogon (Isohelea) sp. 2ES	adult	FinLoc65	24-Jun-2010	Ekrem et al.	KJ767922
FiCer141	Brachypogon (Isohelea) sp. 2ES	adult	FinLoc65	20-Jul-2010	Ekrem et al.	KJ767923
FiCer235	Brachypogon (Isohelea) sp. 2ES	adult	FinLoc65	10-Aug-2010	Ekrem et al.	KJ767924
FiCer139	Brachypogon (Isohelea) sp. 2ES	adult	FinLoc65	20-Jul-2010	Ekrem et al.	KJ767925
FiCer31	Brachypogon (Isohelea) sp. 2ES	adult	FinLoc65	24-Jun-2010	Ekrem et al.	KJ767926
FiCer12	*Ceratopogon abstrusus*	adult	FinLoc65	24-Jun-2010	Ekrem et al.	KJ767927
FiCer13	*Ceratopogon abstrusus*	adult	FinLoc65	24-Jun-2010	Ekrem et al.	KJ767928
FiCer48	*Ceratopogon grandiforceps*	adult	FinLoc42	02-Jul-2010	Ekrem et al.	KJ767929
FiCer166	*Ceratopogon lacteipennis*	adult	FinLoc65	20-Jul-2010	Ekrem et al.	KJ767932
FiCer136	*Ceratopogon lacteipennis*	adult	FinLoc65	20-Jul-2010	Ekrem et al.	KJ767933
FiCer165	*Ceratopogon lacteipennis*	adult	FinLoc65	20-Jul-2010	Ekrem et al.	KJ767934
FiCer143	*Serromyia femorata*	adult	FinLoc65	20-Jul-2010	Ekrem et al.	KJ768085
FiCer208	*Serromyia femorata*	adult	FinLoc08	23-Jul-2010	Ekrem et al.	KJ768086
Finnmark792	*Probezzia*	pupa	FinLoc92	19-Jun-2010	G.A. Halvorsen	KJ768084
FiCer3	*Bezzia annulipes*	adult	FinLoc65	24-Jun-2010	Ekrem et al.	KJ767873
FiCer61	*Bezzia circumdata*	adult	FinLoc65	08-Jul-2010	Ekrem et al.	KJ767874
FiCer7	*Bezzia rhynchostylata*	adult	FinLoc65	24-Jun-2010	Ekrem et al.	KJ767875
FiCer36	*Bezzia rhynchostylata*	adult	FinLoc65	24-Jun-2010	Ekrem et al.	KJ767876
FiCer11	*Bezzia rhynchostylata*	adult	FinLoc65	24-Jun-2010	Ekrem et al.	KJ767877
FiCer32	*Bezzia rhynchostylata*	adult	FinLoc65	24-Jun-2010	Ekrem et al.	KJ767878
FiCer133	*Palpomyia puberula*	adult	FinLoc05	27-Aug-2010	Ekrem et al.	KJ768069
FiCer85	*Palpomyia remmi*	adult	FinLoc05	23-Jul-2010	Ekrem et al.	KJ768070
FiCer227	*Palpomyia remmi*	adult	FinLoc08	23-Jul-2010	Ekrem et al.	KJ768071
FiCer209	*Palpomyia remmi*	adult	FinLoc08	23-Jul-2010	Ekrem et al.	KJ768072
FiCer207	*Palpomyia remmi*	adult	FinLoc08	23-Jul-2010	Ekrem et al.	KJ768073
FiCer200	*Palpomyia remmi*	adult	FinLoc08	07-Aug-2010	Ekrem et al.	KJ768074
FiCer198	*Palpomyia remmi*	adult	FinLoc05	07-Aug-2010	Ekrem et al.	KJ768075
FiCer87	*Palpomyia remmi*	adult	FinLoc05	23-Jul-2010	Ekrem et al.	KJ768076
FiCer132	*Palpomyia remmi*	adult	FinLoc05	27-Aug-2010	Ekrem et al.	KJ768077
FiCer168	*Palpomyia serripes*	adult	FinLoc65	20-Jul-2010	Ekrem et al.	KJ768078
FiCer92	*Palpomyia serripes*	adult	FinLoc65	20-Jul-2010	Ekrem et al.	KJ768079
FiCer170	*Palpomyia serripes*	adult	FinLoc65	20-Jul-2010	Ekrem et al.	KJ768080
FiCer78	*Palpomyia serripes*	adult	FinLoc65	30-Jul-2010	Ekrem et al.	KJ768081
FiCer169	*Palpomyia serripes*	adult	FinLoc65	20-Jul-2010	Ekrem et al.	KJ768082
FiCer167	*Palpomyia serripes*	adult	FinLoc65	20-Jul-2010	Ekrem et al.	KJ768083
FiCer218	Ceratopogonidae gen. sp. 1ES	larva	FinLoc07	14-Jun-2011	T. Ekrem, E. Stur	KJ767930
FiCer217	Ceratopogonidae gen. sp. 1ES	larva	FinLoc07	14-Jun-2010	T. Ekrem, E. Stur	KJ767931
